# Three surgical approaches for retroperitoneal transvaginal natural orifice transluminal endoscopic surgery (vNOTES)

**DOI:** 10.3389/fmed.2026.1780428

**Published:** 2026-04-22

**Authors:** Lu Huang, Xiong Xiao, Dan Feng, Yan Li, Qiannan Hou, Li He, Yonghong Lin

**Affiliations:** Department of Gynecology, Chengdu Women’s and Children’s Central Hospital, School of Medicine, University of Electronic Science and Technology of China, Chengdu, Sichuan, China

**Keywords:** retroperitoneal surgery, transvaginal natural orifice transluminal endoscopic surgery, vaginal fornix approaches, vNOTES, vNOTES lateral suspension, vNOTES lymph node dissection, vNOTES sacrocolpopexy, vNOTES sacrospinous ligament fixation

## Abstract

**Objective:**

This study aimed to evaluate the safety, efficacy, advantages, and limitations of three surgical approaches performed through the anterior, lateral, and posterior vaginal fornix for retroperitoneal transvaginal natural orifice transluminal endoscopic surgery (vNOTES).

**Method:**

We included women who underwent vNOTES retroperitoneal surgery between January 2019 and September 2025. The anterior, lateral, and posterior vaginal fornix approaches were used for retroperitoneal vNOTES. Demographic data, operative characteristics, and perioperative outcomes were retrospectively evaluated.

**Results:**

A total of 89 patients successfully underwent vNOTES retroperitoneal surgery. The surgical approaches included 13 procedures via the anterior fornix, 16 via the lateral fornix, and 60 via the posterior fornix. The anterior fornix approach was primarily used for lateral suspension for pelvic organ prolapse (POP). All procedures were completed safely, with the longest follow-up exceeding 2 years, and no cases of mesh exposure were observed. Overall, three patients (23.08%) experienced Grade I complications. The lateral fornix approach was primarily used for lymphadenectomy, and all procedures were completed safely. A total of four cases (25.00%) of Grade I complications and one case (6.25%) of Grade II complications were recorded. The posterior fornix approach was primarily used for sacrocolpopexy and sacrospinous ligament fixation (SSLF), with all procedures completed safely. In the sacrocolpopexy group, three Grade I complications (30.77%) and one Grade III complication (7.69%) were observed. In the SSLF group, eight Grade I complications (17.02%) were recorded.

**Conclusion:**

In this retrospective study, retroperitoneal vNOTES was demonstrated to be a safe and feasible approach. By using different vaginal fornix incisions, it can be adapted to a wide range of procedures, including surgeries for POP and resection of both pelvic and para-aortic lymph nodes.

## 1 Introduction

The retroperitoneal approach is a surgical technique that involves operating within the space between the peritoneum and the abdominal wall, without entering the peritoneal cavity. Currently, it is predominantly used in urological procedures, retroperitoneal tumor resections, and retroperitoneal lymph node dissections (1–4). The retroperitoneal route offers several distinct advantages. First, the retroperitoneal space is composed of loose connective tissue with relatively sparse vascularity. Through careful dissection, a clear operative field with minimal bleeding can be achieved. Second, as this area is not obstructed by intestinal loops or the greater omentum, surgical exposure is substantially enhanced, particularly in obese patients (5). Third, in patients with a history of pelvic surgery or severe adhesions due to chronic pelvic inflammatory disease, the retroperitoneal approach allows surgeons to bypass adhesions and access the target area directly, thereby reducing the risk of iatrogenic injury. Finally, since the procedure does not involve entering the abdominal cavity, the peritoneum remains intact. This minimizes the impact on the intra-abdominal environment and helps prevent postoperative peritoneal adhesions (6).

Natural orifice transluminal endoscopic surgery (NOTES) represents a cutting-edge advancement in minimally invasive surgery. Its core concept involves using natural orifices, such as the mouth, the vagina, or the rectum, to establish surgical access, thereby achieving scar-free outcomes on the body surface. In gynecology, transvaginal NOTES (vNOTES) has garnered significant attention due to its unique anatomical advantages. This technique involves creating a surgical pathway through a vaginal incision, integrating the principles of conventional vaginal surgery with single-port laparoscopy to perform a variety of gynecological procedures. Previous studies have shown that vNOTES not only avoids abdominal wall incisions, offering improved cosmetic results, but also reduces postoperative pain and accelerates patient recovery (7–9). Within the vNOTES technical framework, the retroperitoneal approach has emerged as an innovative surgical route, demonstrating distinct clinical advantages. In current gynecological practice, retroperitoneal vNOTES is primarily employed for lymph node dissection and procedures related to pelvic organ prolapse (POP) (10, 11). Vaginal incisions are mainly made in the lateral fornix or the posterior fornix, while the anterior fornix approach is less commonly used. The lateral fornix incision is predominantly used for lymph node dissection (12), whereas the posterior fornix approach is mainly used in sacrocolpopexy (13) and sacrospinous ligament fixation (SSLF) (14). There are sporadic reports of the anterior fornix approach being used for lymph node dissection (15) and lateral suspension procedures (16).

However, several important questions remain: What are the differences between these three surgical approaches? How do they compare in terms of safety and efficacy? Which types of surgeries are each approach best suited for? What are their respective advantages and limitations? Currently, no studies have systematically addressed these questions. Therefore, we conducted a retrospective analysis of medical records from our hospital involving retroperitoneal vNOTES procedures performed through the anterior, lateral, and posterior fornix approaches to evaluate and compare the safety, efficacy, advantages, and limitations of these three surgical routes.

## 2 Materials and methods

### 2.1 Patients

We retrospectively analyzed the data of patients in our hospital who underwent retroperitoneal vNOTES surgery between January 2019 and September 2025. This retrospective study was approved by the Institutional Review Board of Chengdu Women’s and Children’s Central Hospital [No. 2025 (129)]. The surgeries were performed by YoL and LHe, who had extensive experience in vNOTES surgery.

Demographic data and medical histories of the patients, including age, body mass index (BMI), previous vaginal delivery, and history of pelvic or abdominal surgery, were obtained from the hospital database and patient files. Perioperative data, including operative time, intraoperative blood loss, intraoperative complications, postoperative Visual Analog Scale (VAS) pain scores at 12 h and 24 h, time to first flatus (days), postoperative stay (days), and complications during hospitalization, were recorded. Complications were scored according to the Clavien–Dindo classification.

### 2.2 Surgical technique

#### 2.2.1 Anterior vaginal fornix approach

**Figure 1:**
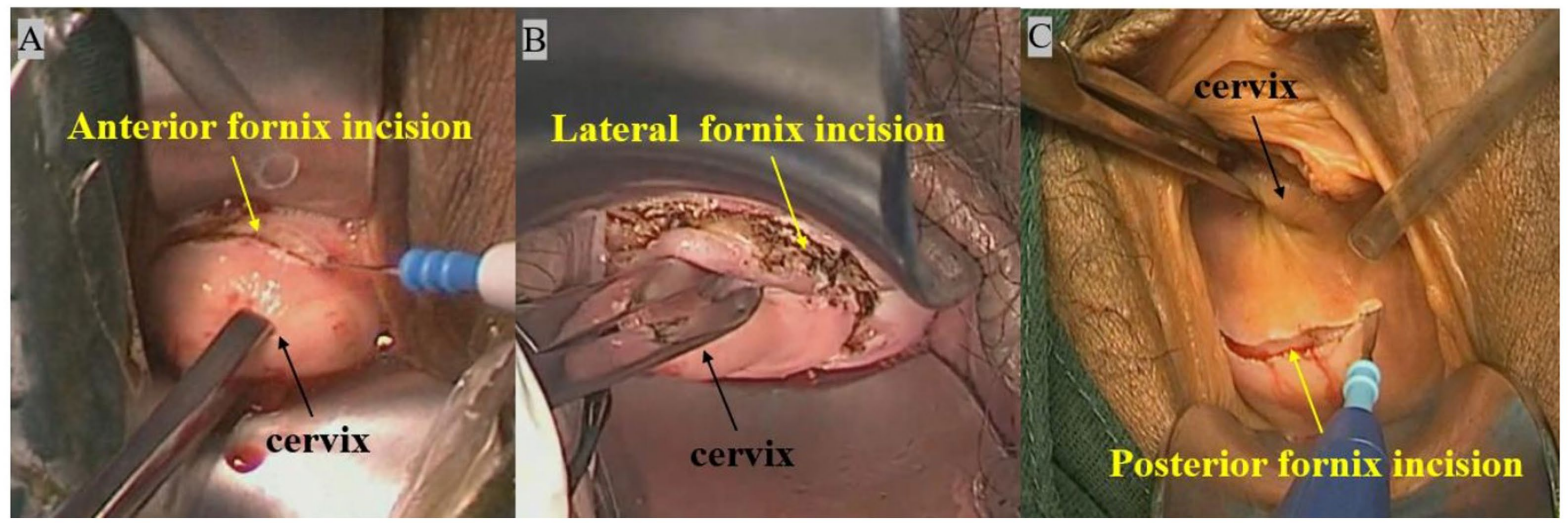
**(A)** Anterior vaginal fornix incision. **(B)** Lateral vaginal fornix incision. **(C)** Posterior vaginal fornix incision.

A 3-cm anterior vaginal mucosa incision was made horizontally between the inferior margin of the bladder and the cervicovaginal junction (Figure 1A). This incision was similar to the traditional incision used to initiate a transvaginal hysterectomy. Using a combination of sharp and blunt dissection, the bladder was dissected away from the vaginal mucosa. Then, blunt dissection was performed laterally with the index finger until the space between the anterior and posterior lobes of the broad ligament was entered. A disposable multi-instrument access port (HK-TH-60.4TY; Beijing Aerospace Kadi Technology Development Institute, China) was inserted into the space, and 14 mmHg of CO_2_ was insufflated. Following laparoscopic instrument insertion (10-mm, 30° endoscope; Karl Storz GmbH & Co. KG, Tuttlingen, Germany), the retroperitoneal space was further dissected cephalad and laterally to create a pathway for mesh (TiLOOP^®^ Total 4, pfm medical titanium gmbh, Nuremberg, Germany) placement (Supplementary Video S1). This approach was commonly employed in the vNOTES lateral suspension for POP at our hospital.

#### 2.2.2 Lateral vaginal fornix approach

Following the induction of general anesthesia, indocyanine green (ICG) was injected into the cervix at the 3 o’clock and 9 o’clock positions for sentinel lymph node mapping. At each position, both deep (1.5 cm) and superficial (0.5 cm) injections were administered. Each injection consisted of 1 mL of a 1.25 mg/mL ICG solution. A 3-cm lateral vaginal fornix incision was created (Figure 1B). Lateral dissection was performed using the index finger to develop the paravesical space. A disposable multi-instrument access port was inserted into the space, and 14 mmHg of CO_2_ was insufflated. Using standard laparoscopic instruments, the lateral pelvic retroperitoneal space was further developed. Blunt dissection was performed in the medial and ventral directions, carefully pushing the bladder and parietal peritoneum to enlarge the pelvic retroperitoneal space and identify pelvic anatomical structures. Under the pressure of carbon dioxide gas, blunt instruments could be separated more easily. It was easy to identify the obturator nerve by separating the lateral pelvic retroperitoneal space, which is a commonly used route for vNOTES pelvic lymph node resection (Supplementary Video S2).

#### 2.2.3 Posterior vaginal fornix approach

A 3-cm vaginal posterior fornix incision was made transversely without opening the peritoneum (Figure 1C). Then, blunt dissection was performed with the index finger to develop the right pararectal space. A 24-Fr double-lumen balloon catheter was positioned within this space, and the balloon was inflated with saline to further dissect the space between the right uterosacral ligament and the rectum, thereby establishing an extraperitoneal space. A disposable multi-instrument access port was then inserted into this space, followed by insufflation with CO₂ to maintain a pressure of 14 mmHg. In sacrocolpopexy, standard laparoscopic instruments were used to extend the pelvic retroperitoneal space cephalad until the sacral promontory was exposed, thereby creating a pathway for mesh (TiLOOP^®^ Mesh light, pfm medical titanium gmbh, Nuremberg, German) placement (Supplementary Video S3). For SSLF, the procedure was largely similar to the aforementioned steps. Under the direct guidance of a laparoscope, we continued to separate the paravaginal space until the sacrospinous ligament was revealed, and in this process, due to the lack of anatomical indication, we placed a finger into the rectum to indicate the ischial spine, if necessary. The sacrospinous ligament was sutured in a figure-eight pattern at the point 1.5–2 cm medial to the ischial spine with a #1 Surgilon-coated braided nylon non-absorbable suture. Although this pathway also enables access to the lateral pelvic wall and exposure of the retroperitoneal vasculature for pelvic lymphadenectomy, its primary application remains in vNOTES sacrocolpopexy and SSLF.

## 3 Results

A total of 89 patients successfully underwent vNOTES retroperitoneal surgery. The surgical approaches included 13 patients via the anterior fornix, 16 via the lateral fornix, and 60 via the posterior fornix. The patients’ baseline characteristics and surgical outcomes are detailed in Table 1.

**Table 1:** Characteristics of patients and surgical outcomes.

Characteristics of patients	Anterior vaginal fornix approach	Lateral vaginal fornix approach	Posterior vaginal fornix approach	
	Lateral suspension for POP (*n* = 13)	Pelvic lymph node resection (*n* = 16)	Sacrocolpopexy (*n* = 13)	Sacrospinous ligament fixation (*n* = 47)
Age ( $\bar{x}\pm s$ , years)	52.08 ± 4.77	53.13 ± 11.89	51.31 ± 7.55	61.09 ± 7.94
BMI ( $\bar{x}\pm s$ , kg/m^2^)	24.22 ± 2.98	22.96 ± 2.26	25.98 ± 3.08	24.49 ± 3.03
Parity ( $\bar{x}\pm s$ , times)	1.46 ± 0.69	1.63 ± 0.72	1.69 ± 0.75	1.64 ± 0.87
Vaginal birth (*n*, %)	13 (100)	16 (81.25)	13 (100)	47 (100)
History of previous pelvic surgery (*n*, %)	7 (53.85)	7 (43.75)	6 (46.15)	8 (17.02)
Preoperative POP-Q stage for apical compartment prolapse (*n*, %)				
I	—	—	—	3 (6.38)
II	4 (30.77)	—	1 (7.69)	11 (23.41)
III	9 (69.23)	—	7 (53.85)	30 (63.83)
IV	—	—	5 (38.46)	3 (6.83)
Operation time ( $\bar{x}\pm s$ , min)	148.62 ± 36.56	160.75 ± 39.31	165.77 ± 35.17	131.79 ± 33.36
Estimated blood loss ( $\bar{x}\pm s$ , mL)	50 ± 17.80	150.63 ± 169.45	196.92 ± 224.48	82.09 ± 34.78
Hemoglobin decreases 72 h post-operation ( $\bar{x}\pm s$ , g/L)	19.85 ± 8.26	29 ± 11.97	22.77 ± 11.18	18.21 ± 9.15
Postoperative stay ( $\bar{x}\pm s$ , days)	3.85 ± 1.52	6.88 ± 3.05	4.07 ± 1.66	4.64 ± 1.13
Time of flatus after surgery ( $\bar{x}\pm s$ , h)	11.23 ± 4.62	13.69 ± 7.15	10.00 ± 4.22	11.15 ± 4.14
VAS scores 12-h post-operation ( $\bar{x}\pm s$ )	2.07 ± 0.76	2.75 ± 0.45	2.54 ± 0.52	2.49 ± 0.55
VAS scores 24-h post-operation ( $\bar{x}\pm s$ )	1.69 ± 0.63	2.13 ± 0.72	1.77 ± 0.73	1.51 ± 0.55
Complications (*n*, %)				
None	10 (76.92)	11 (68.75)	8 (61.54)	39 (82.98)
Grade I	3 (23.08)	4 (25.00)	4 (30.77)	8 (17.02)
Grade II	0	1 (6.25)	0	0
Grade III-a	0	0	1 (7.69)	0
Endometrial histology and stage (*n*, %)				
Endometrioid	—	15 (93.75)	—	—
Clear cell	—	1 (6.25)	—	—
International Federation of Gynecology and Obstetrics (FIGO) stage (2023)				
IA 1	—	5 (31.25)	—	—
IA2	—	7 (43.75)	—	—
IB	—	1 (6.25)	—	—
IIB	—	2 (12.50)	—	—
IIIA1	—	1 (6.25)	—	—
Grading				
1	—	9 (56.25)	—	—
2	—	4 (25.00)	—	—
3	—	3 (18.75)	—	—

The anterior fornix approach was primarily used for POP lateral suspension. All procedures were completed safely, with the longest follow-up period exceeding 2 years, and no cases of mesh exposure were observed. Overall, three patients (23.08%) experienced Grade I complications: one case of acute glaucoma that improved with medication, one case of urinary retention that resolved after 1 week of catheterization, and one case of postoperative cough that improved with antitussive treatment.

The lateral fornix approach was primarily used for lymphadenectomy, and all procedures were completed safely. A total of four cases (25.00%) of Grade I complications were recorded, all manifesting as postoperative fever, which resolved following antipyretic medication. One case (6.25%) of Grade II complications involved postoperative anemia that improved after blood transfusion therapy.

The posterior fornix approach was primarily used for sacrocolpopexy and SSLF, with all procedures completed safely. In the sacrocolpopexy group, three Grade I complications (30.77%) occurred: two cases of fever that resolved with antipyretic medication and one case of urinary retention that resolved after 1 week of catheterization. One Grade III complication (7.69%) was observed involving vaginal mesh exposure, which required surgical excision. In the SSLF group, eight Grade I complications (17.02%) were recorded: six cases of postoperative fever that improved with antipyretics and two cases of urinary retention that resolved after 1 week of catheterization.

## 4 Discussion

Our findings indicate that the three vNOTES retroperitoneal approaches are safe and feasible. The anterior fornix approach is primarily used for lateral suspension in POP, the lateral fornix approach is mainly used for pelvic and para-aortic lymphadenectomy, and the posterior fornix approach is chiefly employed for sacrocolpopexy and SSLF. All surgical approaches successfully reached the target tissues without causing any intraoperative organ injuries.

We believe that the successful implementation of these three pathways primarily stems from the support of the concept of membrane anatomy. “Membrane anatomy” refers to a surgical approach based on the mesenteries formed during the embryonic development of tissues and organs, along with the adjacent fasciae and their derived heterogeneous fused fascial spaces. Sharp dissection using energy devices within these spaces is particularly suitable for organ resection or the reconstruction of normal anatomical positions under the magnified sub-microscopic view provided by laparoscopy (17, 18). The retroperitoneal space is an example of such a heterogeneous, fused fascial space, a potentially expandable space formed during the embryonic stage at the interface of organ mesenteries. Within this space, loose connective tissue exhibiting a filamentous connection pattern can be observed, presenting under laparoscopy as the “angel’s hair” characteristic of membrane anatomy. Procedures performed in this space allow for bloodless dissection. Compared to intraperitoneal surgery, this approach provides more precise anatomy, reduced bleeding, and the effective and safe removal of pathological lesions, ultimately optimizing patient treatment and recovery outcomes (19).

The surgical approach via the lateral vaginal fornix in vNOTES is primarily used for pelvic and para-aortic lymph node dissection. Previous studies (20) reported that the sentinel lymph node mapping of endometrial cancer via the vNOTES lateral fornix approach had an operative time of 126 ± 45.14 min, whereas in our study it was 160.75 ± 39.31 min. Intraoperative blood loss was 78.57 ± 142.18 mL, compared with 150.63 ± 169.45 mL in our study. We believe that the longer operative time and greater blood loss in our study may be attributed to the higher stage of endometrial cancer in our cases, which likely increased surgical difficulty (their study included four cases of endometrial cancer and three cases of atypical endometrial hyperplasia). Although the lateral vaginal fornix approach involves two incisions, it provides easy access to the obturator space, which is highly advantageous for lymph node dissection. Moreover, the direction of vNOTES lymph node removal follows the natural drainage pathway of the uterine lymphatics from caudal to cranial, reducing the risk of accidental resection of non-sentinel lymph nodes. Although this is opposite to the direction of transabdominal laparoscopic surgery, this technique similarly enables complete lymph node mapping from caudal to cranial. Furthermore, the transvaginal approach offers a shorter route to the sentinel lymph nodes, significantly reducing interference from abdominal adipose tissue during surgical manipulation (20–22). We believe that this approach has the potential to become the standard access route for vNOTES pelvic lymph node dissection. Nevertheless, the dissection path to access the obturator fossa through the lateral vaginal fornix is the same as that used in Schauta’s surgery for cervical cancer, which may pose challenges for surgeons unfamiliar with this technique (23).

Currently, the vNOTES anterior fornix approach has been reported for lateral suspension in POP, demonstrating its procedural safety and feasibility (16, 24). However, the procedure described in these studies was not performed entirely via a retroperitoneal approach. The parietal peritoneum was incised during surgery to enter the pelvic cavity, which does not fully exploit the advantages of a completely retroperitoneal approach. In our study, vNOTES lateral suspension was performed using a pure extraperitoneal approach, without opening the peritoneum to enter the pelvic cavity. After accessing through the anterior fornix, the surgical pathway extends cephalad and bilaterally between the anterior and posterior leaves of the broad ligament. Through this space, the arms of the mesh are passed and exteriorized through the abdominal wall. The entire procedure is performed retroperitoneally, avoiding interference from intra-abdominal organs such as the intestines. Dissection within this loose connective tissue space also reduces the risk of bleeding. In addition, without a peritoneal incision, the risk of mesh exposure is reduced. Furthermore, recent studies (25–27) have demonstrated that the anterior fornix approach can also be used for vNOTES pelvic lymph node dissection. In the described technique, a longitudinal incision of approximately 4 cm in length is made in the anterior vaginal wall mucosa, beginning at the bladder neck and extending cephalad. This incision is similar to the initial approach used in traditional anterior colporrhaphy for cystocele repair. Using a combination of sharp and blunt dissection, the bladder is separated laterally from the vaginal mucosa until the arcus tendineus fascia pelvis (ATFP) is identified. Dissection is then continued along the ATFP toward the internal obturator muscle to access the paravesical space. A disposable multi-instrument access port is placed to establish CO_2_ pneumoperitoneum, facilitating laparoscopic visualization for further dissection and identification of the obturator nerve prior to performing pelvic lymph node dissection. These studies indicate that, although the vNOTES lateral fornix approach has proven effective for lymph node dissection, it poses considerable technical challenges. In contrast, the anterior vaginal fornix approach allows for complete lymph node dissection through a single incision and is considered easier to teach and learn.

The posterior vaginal fornix approach in vNOTES provides comparatively facile access to the retroperitoneal compartment and is commonly used in surgical procedures for POP. Contemporary studies (11, 13, 28–30) have reported on vNOTES sacrocolpopexy; however, the majority of these reports involve opening the peritoneum to access the pelvic cavity for suturing. Only one study has described uterus-preserving retroperitoneal vNOTES sacral hysteropexy, whereas all procedures in our study were performed using a purely retroperitoneal approach. The mesh fixation is completed within the retroperitoneal space without incising the pelvic peritoneum, thereby theoretically mitigating the risk of subsequent mesh exposure. Additionally, the “bottom-up” visualization afforded by vNOTES provides greater exposure of the presacral region than traditional laparoscopy, which may reduce the risk of iatrogenic injury to vascular and neural structures. The same approach is used for vNOTES SSLF. Crucially, while traditional transvaginal SSLF is conducted retroperitoneally, it depends on largely blind maneuvers, with suture placement guided by palpation of the ischial spine. vNOTES, by enabling direct laparoscopic visualization, permits precise suture placement under direct sight, which is anticipated to enhance procedural accuracy and contribute to superior anatomical success rates (14, 31). Currently, there is a report of one successful case of vNOTES SSLF (31). Our team’s previous report also demonstrated that the posterior vaginal fornix approach is safe and feasible for SSLF (14).

## 5 Conclusion

In this retrospective study, retroperitoneal vNOTES was demonstrated to be a safe and feasible approach. By using different vaginal fornix incisions, it can be adapted to a wide range of procedures, including surgeries for POP and resections of both pelvic and para-aortic lymph nodes. Further large-sample, multicenter randomized controlled trials are warranted to validate the efficacy, safety, and advantages of these approaches.

## Data Availability

The raw data supporting the conclusions of this article will be made available by the authors, without undue reservation.
